# Bacteriophages inhibit and evade cGAS-like immune function in bacteria

**DOI:** 10.1016/j.cell.2025.08.009

**Published:** 2025-08-22

**Authors:** Erin Huiting, Xueli Cao, Jie Ren, Januka S. Athukoralage, Zhaorong Luo, Sukrit Silas, Na An, Héloïse Carion, Yu Zhou, James S. Fraser, Yue Feng, Joseph Bondy-Denomy

Our study established a naturally active Type II-A CBASS model system in the *Pseudomonas aeruginosa* strain BWHPSA011 (NZ_AXQR00000000.1). In the method details for the section on ‘‘Identification of CBASS operons,’’ there were four errors with the NCBI identifiers:

1. CBASS operon NCBI contig ID was incorrectly indicated as NZ_AXQR000000012.1, and the correct ID is AXQR01000012.1.
2. CapV NCBI nucleotide ID was incorrectly indicated as Q024_30602, and the correct ID is Q024_03602. The corresponding NCBI protein IDs are ERW74313.1 or P0DX85.1.
3. CdnA NCBI nucleotide ID was incorrectly indicated as Q024_30601, and the correct ID is Q024_03602. The corresponding NCBI protein IDs are ERW74312.1 or P0DX86.1.
4. Cap2 NCBI nucleotide ID was incorrectly indicated as Q024_30600, and correct ID is Q024_03600. The corresponding NCBI protein IDs are ERW74311.1 or P0DX87.1.

The method details in this section also provide the nucleotide range for the Cap3 gene because it is not annotated in the published BWHPSA011 genome. For clarity, we have provided the nucleotide and protein sequences of Cap3 below. We apologize for any confusion that these errors may have caused.

Cap3 nucleotide sequence:

ATGCAATTCATGAGTTCTTGGGCGGCCGACGACAACAGAACGCTGCTGCATTTTTCGAAGTCCACGCTGGAGACCTTCCGCCAGC ATGTCCAGGCCAGTGATTCTGACTGCGAAGCTGGCGGGCTGCTACTCGGTTCGGTCCATGGAACTCACATGCTCATTGAGCATGC AACGGTTCCCACGGCATGGGACAAACGGTTTCGCTACCTGTTCGAGAGAATGCCATTCGGCCACGAAGCCATTGCACTGGCTCGAT GGACGGCGAGCCAGGGAACCATCCGCCATTTGGGCGAATGGCACACCCATCCGGAAGACAACCCCAACCCGTCGGGCCTCGATA GATCGGAGTGGAACCGTCTGTCGGCCAAGCGTCGGGATAAGCGACCAACGCTCGCTGTCATTGTTGGCCGAAATGCCCTGTATAT TGAGTTGGTGCCAGGCTCAGGCCAAGGATCGGTATTCTCCCCTGTGGAATAG

Cap3 protein sequence:

MQFMSSWAADDNRTLLHFSKSTLETFRQHVQASDSDCEAGGLLLGSVHGTHMLIEHATVPTAWDKRFRYLFERMPFGHEAIALARWTASQ GTIRHLGEWHTHPEDNPNPSGLDRSEWNRLSAKRRDKRPTLAVIVGRNAL YIELVPGSGQGSVFSPVE*

